# Individual causal attribution in occupational disease claims: a structured epidemiological approach

**DOI:** 10.1186/s13584-026-00752-5

**Published:** 2026-02-27

**Authors:** Eytan Ellenberg, Marc Weber, Aviram Weiss, Eldad Katorza, Akshaya Bhagavathula

**Affiliations:** 1 Office of Medical Affairs, National Insurance Institute of Israel, Jerusalem, Israel; 2 Department of Public Health, College of Health & Human Sciences, North Dakota State University (NDSU), Fargo, ND USA

**Keywords:** Epidemiological causal reasoning, Occupational health, Causal reasoning, Israel, Medico-legal expertise, Evidence-based justice

## Abstract

The determination of whether occupational exposure contributed to disease in an individual worker is a key element in occupational disease adjudication, particularly within public insurance systems such as that of Israel. At present, such assessments often rely on expert judgment grounded in clinical intuition rather than on structured, reproducible inference.This paper proposes a structured epidemiological approach to individual causal attribution, integrating population-based evidence with case-specific analysis. The framework is organised into four analytical stages: appraisal of causal capacity, quantitative estimation of association, individualised causal partitioning, and probabilistic legal interpretation.Israel’s hybrid medico-legal system offers a suitable setting for piloting this approach. Embedding structured epidemiological reasoning into occupational disease adjudication may enhance transparency, scientific integrity, and consistency, thereby strengthening fairness and public trust in compensation decisions.

## Introduction

Causal reasoning in occupational medicine continues to rely heavily on expert judgment, often grounded in physiological plausibility and clinical intuition rather than structured evidence pathways. This commentary does not address whether occupational exposures are capable of causing disease in general, but rather how epidemiological evidence can be translated into transparent and reproducible judgments about causal contribution in individual compensation claims. While historically necessary, this approach has become increasingly inadequate in an era of open data, meta-analyses, and artificial intelligence [1, 2].

n Israel, thousands of occupational disease claims are filed annually, with internal National Insurance Institute data revealing variability in recognition rates across regional committees [3]. Across jurisdictions, medico-legal experts are expected to produce conclusions that are transparent, reproducible, and scientifically defensible—yet the methodological foundations for such expectations remain fragile.

Structured epidemiological reasoning in medico-legal contexts refers to the application of epidemiological methods and causal inference tools to support decision-making in occupational disease adjudication [4]. This approach offers a conceptual and operational bridge between epidemiological science and legal decision-making. It reframes the role of the expert from solitary authority to interpreter of structured evidence. Through this lens, causal attribution becomes a process, not an opinion: a chain of reasoning that can be examined, replicated, and improved.

This paper proposes a four-stage causal reasoning model—critical appraisal of evidence, quantitative risk estimation, multifactorial causal partitioning, and probabilistic legal interpretation—that organizes reasoning into a reproducible, transparent process supported by evidence.

## From the individual case to the population and back

At the heart of Structured epidemiological reasoning in medico-legal contexts lies a dynamic circulation between the individual and the collective. Causal inquiry begins with an individual narrative—a worker, an illness, a suspected exposure—but its validation depends on population-level knowledge. Epidemiology transforms singular experiences into collective evidence, turning stories into data and patterns into probabilities.

**Fig. 1:**
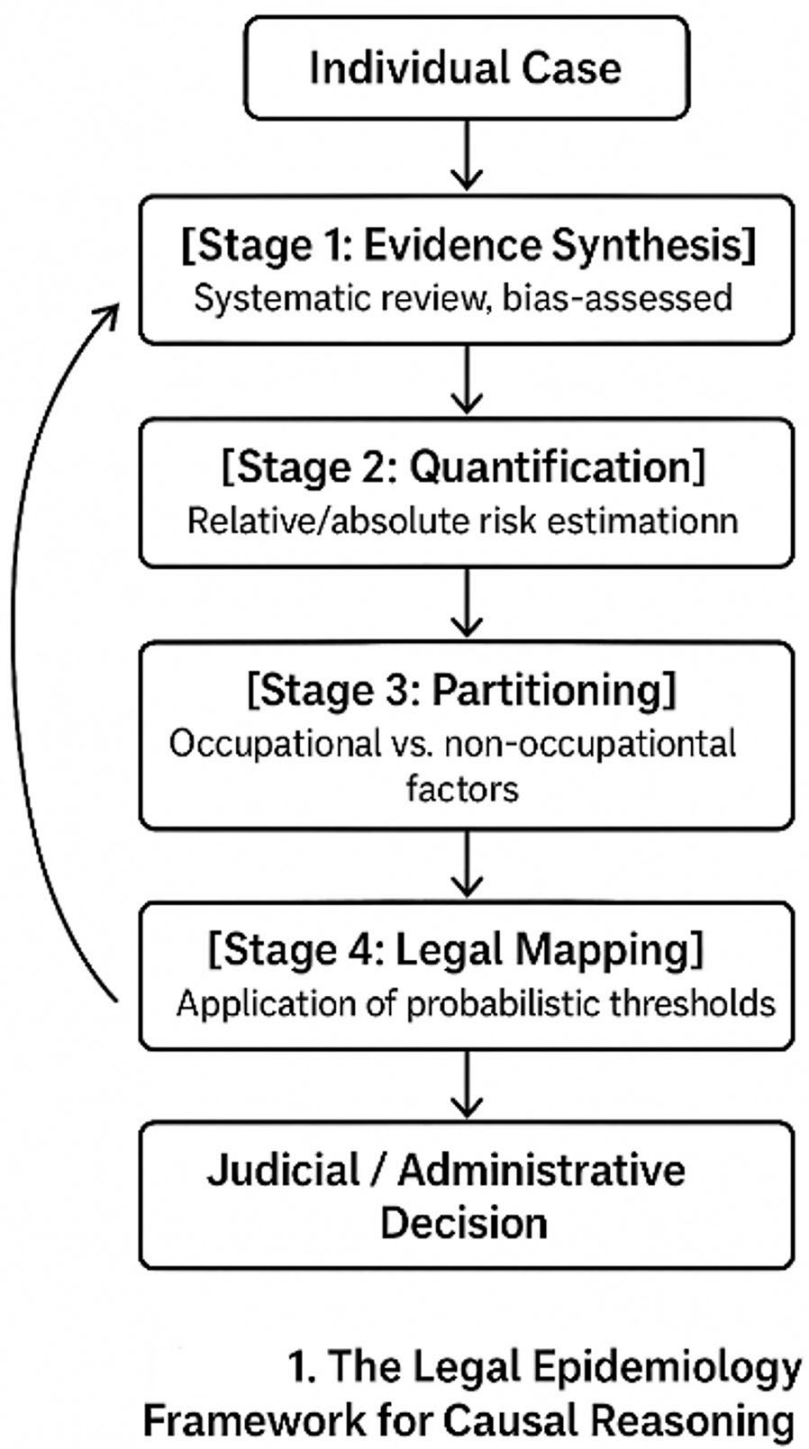
Feedback loop between individual case assessment and population-level inference within the Structured epidemiological reasoning in medico-legal contexts framework, illustrating how structured evidence flows bidirectionally between clinical judgment and epidemiologic data

Yet justice demands a return journey: the aggregated probability must be translated back to the individual case, interpreted in light of personal exposure history, vulnerability, and rights. This back-and-forth movement—from case to population and back again—is not merely procedural; it embodies the epistemological rhythm of modern science. Knowledge grows through iteration between lived experience and statistical abstraction. Structured epidemiological reasoning in medico-legal contexts formalizes this dialogue, ensuring that what is true for populations becomes meaningful and fair for individual. Figure 1 illustrates this bidirectional circulation between individual cases and population-level data.

This schematic represents the circular flow between individual cases and collective evidence through the four analytical stages of Structured epidemiological reasoning in medico-legal contexts, illustrating the iterative feedback loop that connects judicial reasoning with evolving population data.

### Four analytical stages for structured causal reasoning

Structured epidemiological reasoning in medico-legal contexts structures this circulation through four analytical stages that jointly define causal reasoning in occupational medicine:

1. Evidence of causal capacity (population-level appraisal)

This stage evaluates whether high-quality epidemiological evidence supports a causal association between the occupational exposure and the disease. In the absence of such evidence, individual causal attribution is not undertaken. Experts should assess the totality of available studies through systematic, bias-assessed synthesis following PRISMA or OHAT principles [5].

2. Quantitative estimation of association

Measures such as relative risk, odds ratio, and absolute risk difference are extracted from the literature and translated into parameters usable by courts and insurers, expressing the strength and direction of association [6].

3. Individualised causal partitioning

Occupational diseases are rarely monocausal. Quantitative methods—including Bayesian integration or Shapley-value decomposition—permit proportional allocation of causal contributions across occupational and non-occupational factors [7].

Together, Stages 2 and 3 aim to estimate the magnitude of occupational risk as it applies to an individual worker, rather than to an abstract population average.

4. Probabilistic legal interpretation

Quantified evidence is then interpreted in relation to explicit legal standards of proof—such as “more likely than not” (> 50%) or “substantial contribution” (≥ 20%)—thereby connecting epidemiological reasoning with legal responsibility [8].

To illustrate, consider a 58-year-old construction worker with lung cancer following prolonged exposure to crystalline silica. In Stage 1, more than twenty cohort studies are synthesised (IARC Monograph 100 C, 2012 [9], updated by subsequent meta-analyses). Stage 2 yields a pooled relative risk of approximately 1.8 (95% CI 1.5–2.2). Stage 3 partitions causation between occupational exposure (≈ 45%) and smoking history (≈ 55%). Stage 4 maps these results onto Israeli legal thresholds: if a > 50% standard is required, the claim is denied; if a ≥ 20% standard suffices, partial compensation may be justified. The numerical values presented are illustrative and intended to exemplify the reasoning process rather than to constitute adjudicative recommendations.

Together, these stages transform medico-legal evaluation from an art grounded in intuition into an auditable process of causal inference, aligning health and legal decision-making around shared epistemic standards.

Importantly, the legal thresholds used in occupational adjudication correspond to distinct inferential objects. The “more likely than not” standard (> 50%) reflects an assessment of overall causal plausibility, grounded in the totality of epidemiological and clinical evidence, including the strength, consistency, and coherence of associations reported in the literature, as well as their compatibility with the individual clinical history.

By contrast, the “substantial contribution” threshold (≥ 20%) corresponds to a formal causal attribution exercise. In this framework, attribution is derived from explicit mathematical models that quantify excess risk and partition it across multiple causal factors using Shapley-based methods. This approach accounts for joint and competing effects of occupational and non-occupational exposures, avoids double counting of overlapping risks, and respects the underlying multifactorial causal structure. Accordingly, the ≥ 20% threshold is not tied to any fixed relative risk cut-off, but to the estimated share of excess causation attributable to occupational exposure after adjustment for competing pathways.

## The israeli context: a testbed for evidence-based causation

Israel offers a distinctive opportunity to operationalize this methodological refinement. The Bituach Leumi (National Insurance Institute of Israel, NII) system recognizes occupational injuries and diseases but still relies on variable expert assessments of the causal link (Kesher Sibati in Hebrew). Evaluations often rest on experience rather than standardized evidence models, producing heterogeneous outcomes and perceived inequities, as documented in case law variability [10]. 

The hybrid nature of Israel’s medico-legal framework—civil-law foundations combined with strong judicial discretion—creates both flexibility and fragmentation. Recent developments in electronic exposure registries, national health–labor data integration, and emerging AI-assisted analyses in Israeli academic medical centers make it possible to move toward standardized, transparent reasoning.

Structured epidemiological reasoning in medico-legal contexts could thus serve as a scientific infrastructure for justice, harmonizing legal thresholds, medical expertise, and social equity. This evolution aligns with Israel’s broader health-policy goals of reducing disparities, improving decision consistency, and strengthening social trust in medico-legal institutions [11] (Table 1).

**Table 1:** Key Terminology

Term	Definition	Context
Structured epidemiological reasoning in medico-legal contexts	Refers to the application of epidemiological methods and causal inference tools to support decision-making in occupational disease adjudication.	Conceptual foundation of the framework.
Kesher Sibati (causal link)	Hebrew term meaning causal link in Israeli occupational law.	Core medico-legal notion in Israel.
Bitouach Leumi (NII)	Israel’s National Insurance Institute, adjudicating occupational disease claims.	Institutional setting for implementation.
Probabilistic Threshold	Legal standard expressed as probability (e.g., > 50% “more likely than not”, ≥ 20% “substantial contribution”).	Connects epidemiology to legal proof.

## Policy and ethical implications

Embedding Structured epidemiological reasoning in medico-legal contexts in occupational adjudication extends beyond technical accuracy. It redefines fairness as a measurable property of decision systems rather than a moral abstraction [12]. By ensuring that comparable cases receive comparable evaluation, it operationalizes the ethical principle of equality before the law.

Critics may argue that probabilistic thresholds risk mechanizing judgment or undermining clinical expertise. Yet structured reasoning does not replace expertise—it channels it toward transparent, defensible interpretation rather than opaque authority.

Implementation should focus on embedding the principles and methods of Structured epidemiological reasoning in medico-legal contexts within the Bituach Leumi’s medico-legal system and Israel’s national framework for occupational disease recognition.

**At the institutional level (NII):**

- Adopt standardized frameworks for evidence synthesis and bias assessment (PRISMA, OHAT)
- Develop simulation-based causal models that translate population-level data into individual probabilistic assessments [12,13]
- Establish a dedicated Structured epidemiological reasoning in medico-legal contexts and Causal Inference Unit within the Office of Medical Affairs to promote applied research and expert training

**At the national level:**

- Integrate occupational and health data registries for transparent, data-driven decision-making.
- Develop evidence-based guidelines on major occupational exposures (carcinogens, ergonomic risks, psychosocial factors).

**At the international level:**

- Partner with research institutions (e.g., NDSU, Temple University) to build an *international registry of occupational exposures and causal models*.
- Contribute to comparative studies and methodological harmonization across legal systems.

This multilevel integration would make Israel a global reference point for transparent, scientifically grounded causal reasoning in occupational health adjudication.

Probabilistic reasoning also allows protection of workers in zones of scientific uncertainty—avoiding the paradox whereby insufficient evidence leads to denial rather than precaution [11, 12]. Given its compact institutional landscape, Israel could realistically pioneer such reform, demonstrating that justice, efficiency, and scientific rigor are not competing goals but convergent ones.

## Conclusion

The modernization of causal reasoning in occupational medicine requires more than new tools—it demands a new epistemology.

Structured epidemiological reasoning in medico-legal contexts offers that operational improvement, creating a structured dialogue between individual experience and collective evidence, between medicine and law, between probability and justice.

Israel, through its hybrid legal system and advanced data capacities, is uniquely placed to lead this transition.

If successful, this model could inform reforms in other jurisdictions—from the UK’s Industrial Injuries Advisory Council to Canada’s workers’ compensation boards—where similar tensions exist between scientific evidence and legal certainty.

In an era where decision-making systems face demands for transparency and reproducibility, the shift towards structured, evidence-based causal reasoning has become an urgent imperative for both legal and health systems. This is not only a methodological shift—it is an ethical commitment to evidence-based justice. Embedding Structured epidemiological reasoning in medico-legal contexts within Israeli law and the NII’s adjudication framework could directly inform forthcoming procedural reforms, promoting transparent, data-driven, and equitable decision-making.

## Data Availability

No datasets were generated or analysed during the current study.
